# India needs a great sanitary awakening

**DOI:** 10.4103/0019-5278.44699

**Published:** 2008-12

**Authors:** J.P. Majra, A. Gur

**Affiliations:** Department of Community Medicine, K.S. Hegde Medical Academy, Deralakatte, Mangalore, India; 1 Department of Periodontics, A B Shetty Memorial Institute of Dental Sciences, Deralakatte, Mangalore – 575018, India

Dear sir,

I read the article titled “Environmental sanitation: An ignored issue in India” published in the April 2008 issue of your esteemed journal with keen interest. The author has chosen a vital subject that needs to be addressed urgently. Environmental sanitation is indeed still an ignored issue in India. This is evident from the facts available from the report of the National Family Health Survey-3 (NFHS-3), which throws light on some of the household characteristics like the type of house, safe drinking water and sanitation, cooking fuel and overcrowding.[1] It is reported that more than half of the households in India live in kachha/semipucca (14% kachha, 40% semipucca) houses. The proportion of households living in pucca houses is lowest in Manipur (10.7%). In 34% of the households, three to four persons and in 29% of the households, five or more persons share a single room for sleeping. Ninety percent of the rural households and 31% of the urban households use solid fuels for cooking, which generate smoke and unhealthy conditions when inhaled. About 90% of the households that use solid fuels cook on an open fire without diverting the smoke through a chimney. Although 88% of the households have access to an improved source of drinking water, only half the households reported having drinking water on their premises (51% urban, 28% rural). For 37% of the households, it takes up to 30 min and for the remaining 12% more than half an hour to fetch drinking water. Transportation and storage is known to cause contamination of water, but only 33%of the households treat their drinking water to improve its potability. More than half (55%) of the households (74% rural, 17% urban) have no toilet facility, the figure varying from 2% for Mizoram to 81.3% for Chhatisgarh. Only 29% of the households have toilet facilities that are improved and not shared with any other household. The survey about disposal of household (solid and liquid) and animal waste, which form a significant source of illness, is silent. Control of these factors has been responsible for a considerable improvement in the health of people during the past century in the developed countries. On the other hand much of the illness in India is due to poor environmental sanitation, i.e., unsafe drinking water, polluted soil, unhygienic disposal of human excreta and refuse, poor housing, insects and rodents.[2] The National Water Supply and Sanitation Programme was launched as early as 1954 and was supported by the Accelerated Rural Water Supply Programme (1971-72), the International Drinking Water Supply and Sanitation Decade (1981-90), the Rajiv Gandhi National Drinking Water Mission (1986), the Central Rural Sanitation Programme, the Total Sanitation Campaign and the Millennium Development Goals(2000) and similar programs. But, it is evident from facts reported by the NFHS-3 that the sanitary conditions have not changed much. The unsanitary conditions are appalling in India and[3] perhaps India also needs a great sanitary awakening like that which took place in London in the mid-nineteenth century.

## References

[1] International Institute for Population Sciences (IIPS) and Macro International. 2007 (2007). National Family Health Survey (NFHS-3), 2005–06: India.

[2] Park K, Environment and health (2007). Park's text book of preventive and social medicine.

[3] Sundarlal, Adarsh, Pankaj (2007). Text book of community medicine.

